# A Fuzzy Logic System for Seizure Onset Detection in Intracranial EEG

**DOI:** 10.1155/2012/705140

**Published:** 2012-03-28

**Authors:** Ahmed Fazle Rabbi, Reza Fazel-Rezai

**Affiliations:** Department of Electrical Engineering, University of North Dakota, Grand Forks, ND 58202, USA

## Abstract

We present a multistage fuzzy rule-based algorithm for epileptic seizure onset detection. Amplitude, frequency, and entropy-based features were extracted from intracranial electroencephalogram (iEEG) recordings and considered as the inputs for a fuzzy system. These features extracted from multichannel iEEG signals were combined using fuzzy algorithms both in feature domain and in spatial domain. Fuzzy rules were derived based on experts' knowledge and reasoning. An adaptive fuzzy subsystem was used for combining characteristics features extracted from iEEG. For the spatial combination, three channels from epileptogenic zone and one from remote zone were considered into another fuzzy subsystem. Finally, a threshold procedure was applied to the fuzzy output derived from the final fuzzy subsystem. The method was evaluated on iEEG datasets selected from Freiburg Seizure Prediction EEG (FSPEEG) database. A total of 112.45 hours of intracranial EEG recordings was selected from 20 patients having 56 seizures was used for the system performance evaluation. The overall sensitivity of 95.8% with false detection rate of 0.26 per hour and average detection latency of 15.8 seconds was achieved.

## 1. Introduction

Epilepsy is the most common neurological disorder which affects 1–3% world's population [1–3]. It is characterized by the occurrence of two or more unprovoked epileptic seizures which are abnormal rhythmic discharge of electrical activity of the brain [1–6]. A seizure is defined as a paroxysmal alteration of one or more neurological functions such as motor, behavior, and/or autonomic functions [1]. Epileptic seizures are episodic, rapidly evolving temporary events. Typically, the duration of epileptic seizure is less than a minute [1–3]. Though the mechanism behind epileptic seizure is not completely known yet, a seizure event can be described as the increased network excitation of the neural networks with synchronous discharge as well as variable propagation in brain [1, 2]. In focal epilepsy, ictal manifestations may localize in a specific brain region, whereas in generalized epilepsy the whole brain could be candidate for seizure events [1, 2].

 Electroencephalogram (EEG) is the most widely used measure for diagnosis of neurological disorders such as epilepsy in clinical settings. Long-term monitoring of EEG is one of the most efficient ways for diagnosis of epilepsy by providing information about patterns of brain electrical activity, type, and frequency of seizures, and seizure focus laterality [1–3, 7]. In long-term monitoring, ictal EEG recording is usually correlated with the clinical manifestation of seizure. If the recording site is where the seizure focus is located, the changes in EEG can occur before the clinical manifestations [1, 2]. In the case that electrodes are placed in remote location from the seizure onset site, the clinical manifestations may occur before any visual changes in EEG. Therefore, the placement of electrodes is a determining factor in seizure detection or early detection [3]. The experts monitoring long-term EEG recordings usually look for earliest visually apparent changes in EEG to identify ictal onset [1]. This information helps physician or caregiver to treat patients early in time with the available medications. However, the visual inspection of long-term EEG by clinicians is challenging since it is performed over several days to weeks due to the unknown nature of time of occurrence of seizures. The visual inspection of this large amount of data to identify seizure is very time consuming and monotonous as well [2–4, 7]. Therefore, an automatic seizure detection tool with high detection rate and considerably low false detection rate would have valuable application in clinical settings in epilepsy treatment [1–4, 7].

 During a seizure event, increased abnormal synchronous firing occurs in the involved neural networks of brain. The pattern and shape of ictal EEG varies according to the brain region as well as types of recordings (intracranial or scalp EEG). A detection algorithm should be able to identify these dynamic changes in EEG with high sensitivity. One of the most common patterns found in ictal EEG is periodic sharp activity (6–8 Hz activity of a mesial temporal lobe-onset seizure) [1, 2]. The ictal onset and offset is also characterized by relatively high complexity signals. However, the ictal initiation patterns may vary from patient to patient. Though the patterns in different patients may vary depending on the type of seizures, proximity of the recording electrodes to the seizure onset site, types of recordings the ictal onset patterns, and early evolution of brain dynamics in a given patient are of similar types. Therefore, the algorithm parameters can be tuned in a patient-specific way to increase the specificity and sensitivity of detections [2, 3]. 

 One of the applications of automatic seizure detection in clinical settings is to monitor patients and localize brain regions. As for medically intractable focal epilepsy, brain tissue of seizure focus is candidate of surgery and the source localization information helps neurologists in surgical procedure [1–3, 7]. Moreover, to provide patients an alternative to surgical treatment, much focus has been put on early detection or prediction of seizure providing sufficient time of intervention prior to clinical onset and ultimately preventing or controlling epilepsy [3]. In theory of early detection, the ictal manifestation in EEG is expected to be detected several seconds to a few minutes earlier [2, 3]. Although the intervention time is crucial in designing a control device, an early detection tool capable of detecting seizure several minutes prior to clinical seizure onset would help the patients in avoiding serious injuries by taking proper action or using available medication to soothe the intensity of seizure frequency [3, 7]. 

 Significant progress has been made in automatic detection of seizure in iEEG over the last couple of decades [2–4, 7–15]. Qu and Gotman [7] developed an automatic seizure detection method to detect various types of seizures in both surface and intracranial EEGs. It was based on decomposition of EEG into elementary waves and detecting paroxysmal bursts of rhythmic activities using relative amplitude, their duration, and rhythmicity [7]. Murro et al. [8] developed a computerized method to detect complex partial seizures. The method used three EEG features, relative amplitude, dominant frequency, and rhythmicity. Discriminant analysis was used for decision making [8]. In order to reasonably reduce the false alarm rate, Qu and Gotman [9] developed a warning system based on template matching which relies on availability of one sample seizure for subsequent detections of similar seizures in scalp and intracerebral EEG recordings. Later, Qu and Gotman [10] proposed a seizure onset detection system with high sensitivity and very low false positive rate. Osorio et al. [11] proposed an algorithm for real-time detection, quantitative analysis of seizures, and prediction of the clinical onsets. Grewal and Gotman [12] proposed an automatic seizure warning system for clinical use. Spectral features were extracted after filtering EEG in multiple bands and Bayes' theorem was used along with spatio-temporal analysis. Though the system requires training in order to obtain the prior probabilities, no patient training is required at run time [12]. In a different approach, Adeli et al. [13] performed wavelet sub-band analysis of EEG and five EEG bands as well as nonlinear analysis of EEG for detecting seizure and epilepsy. They used correlation dimension and largest lyapunov exponent to quantify nonlinear dynamics of EEG [13]. Ghosh-Dastidar et al. [14] proposed a novel wavelet-chaos-neural network methodology for detecting epileptic seizures. Srinivasan et al. [4] proposed a neural network-based automatic seizure detection system using approximate entropy (ApEn) as the input feature. Gardner et al. [5] discussed a one-class support vector machine (SVM) novelty detection for seizures in iEEG by classifying short-time, energy-based statistics. The detector was validated on a sample of 41 interictal and 29 ictal epochs and yielded 97.1% sensitivity, mean detection latency of −7.58 seconds, but false positive rate (FPR) of 1.56 false positive per hour [5]. Chan et al. [6] proposed a patient-specific algorithm for accurate measurement of seizure onset time detection. The algorithm makes use of spectral and temporal features and support vector machine as classifier [6]. Ghosh-Dastidar and Adeli [16] presented a new supervised learning algorithm for Multispiking Neural Networks (MuSpiNN) which was applied in seizure detection. They have demonstrated better accuracy of MuSpiNN over single-spiking Spiking Neural Network (SNN) model [16]. In a recent work, Zhang et al. [17] proposed a novel incremental learning scheme based on nonlinear dimensionality reduction for automatic seizure onset detection. They used continuous wavelet transform (CWT) for feature extraction and two-stage decision making which makes use of nonlinear dimensionality reduction and incremental learning schemes [17].

 Recently, much focus has been put in detection of seizures early in time or eventually predicting it. There have not been much significant works performed in the area of seizure detection or early detection based on fuzzy logic approaches. Subasi [15] introduced the application of adaptive neurofuzzy inference system (ANFIS) for epileptic seizures detection and classification for normal and epileptic patients. This method combined the adaptive capabilities of artificial neural networks and qualitative approach of fuzzy logic and features were extracted using the wavelet transform (WT) [15]. Aarabi et al. [18] presented an automatic method which uses fuzzy rule-based system to detect seizures in iEEG. Temporal, spectral, and complexity features extracted from iEEG were fed into two-stage decision-making systems where they were spatial-temporally integrated. Intermediate decision making was performed in the first stage using rule-based fuzzy inference system. Final decision was made using spatial combiner, feature combiner, and postprocessor [18].

 In the area of seizure prediction and/or early detection, several problems or pitfalls have been identified which requires to be addressed properly and carefully in order to make further progress [19]. Most of the methods available in the literature use single-feature extraction method followed by a predefined crisp threshold for final decision making [19]. Nonlinear methods are popular to most of the researchers; however, most of these methods are sensitive to noise which may lead to wrong findings [18, 19]. Therefore, the advantages of nonlinear feature extraction methods over linear methods are yet to be justified [19]. The selection of test dataset is also critical because direct comparisons of different studies or approaches are difficult unless those are applied to the same dataset [19]. Proper statistical validation remains another major concern [19, 20]. To address one of these challenges, Feldwisch-Drentrup et al. [21] described a method using logical “AND” and “OR” combinations in order to combine two epileptic seizure prediction methods. The study shows improved performance for both the combinations, and the “AND” combination yielded highest sensitivity [21]. In this study, we have applied fuzzy algorithms for combining more than two methods (four in this paper) for seizure onset detection. We utilized fuzzy “AND” combination instead of logical “AND” combination to study the feasibility of this method in early detection. The results show that this approach could be a promising solution to address some of the challenges in the area of early seizure detection and eventually seizure prediction.

 In this paper, we present a fuzzy rule-based adaptive automatic seizure onset detection method. The overall method consists of several steps, preprocessing, artifacts detection, feature extraction, decision making using fuzzy logic, and postprocessing. Time domain, frequency domain, and entropy-based features are extracted from EEG segments. These features are combined using a set of fuzzy rules and another set of fuzzy rules are used to combine information spatially. Final decision was made by applying a threshold procedure to this spatial-temporal combination of multiple features. Artifacts detection algorithm was applied prior to feature extraction to identify segments corrupted with electrode movement and saturation artifacts. The information was stored to be used in postprocessing step. False detections caused by artifacts and other activities were rejected in the postprocessing steps.

## 2. Materials and Methods

### 2.1. EEG Recordings

The iEEG recordings were obtained from the Freiburg Seizure Prediction EEG (FSPEEG) database [22, 23]. The database contains iEEG data from 21 patients with medically intractable focal epilepsies. The sampling frequency of the data is 256 Hz. The database contains six channels with common reference, three located on the epileptogenic zone and three in remote locations [22, 23]. In this study, we selected iEEG datasets obtained from 20 patients to evaluate the performance of the proposed method. The total length of the data analyzed was 112.45 hours and total numbers of analyzed seizures were 56. The details of the iEEG data used in this study are shown in Table 4.

### 2.2. Preprocessing

#### 2.2.1. Segmentation

The multichannel iEEG data were segmented using a moving window analysis technique. The length of each segment was 2.5 seconds (640 data points) with overlap of 0.5 seconds (128 data points) between the adjacent windows along the whole iEEG recording. This window length was chosen as a way to divide the signals into quasistationary segments for correct computation of the characteristic features [24].

#### 2.2.2. Artifacts Detection

Although iEEG data are usually less corrupted with artifacts comparing to scalp EEG, visual inspection confirmed the presence of saturation and electrode movement artifacts in some patient's data. The data files obtained from the FSPEEG database also provide some information on artifacts, mostly movement artifacts and visual inspection was performed based on that information. We implemented an artifacts detection algorithm to identify the EEG segments corrupted with these two types of artifacts: saturation and electrode movement. Each segment with artifacts was marked and the information is stored in memory to be used later in the postprocessing step. The artifacts detection algorithm steps are discussed in following sections.

**(A) Saturation Artifact**

There were several cases of iEEGs corrupted with saturation artifacts. At the saturation time, iEEG signals have constant amplitude. The segments with saturation artifacts were identified by a derivative method. Every segment with zero derivatives was marked as segments with saturation artifacts [18]. A median filter of window size 5 was used to remove all single-segment saturations. This prevents false detection of artifacts in other EEG segments rather than in the region of saturation.

**(B) Electrode Movement Artifact**

Electrode movement artifacts are usually caused by patient's head movement or displacement of the electrode box. This type of artifact is of high amplitude with an upstroke [18]. Analytical signal processing approach was utilized in order to detect envelope of iEEG segments using Hilbert transform [25]. Average absolute envelope (*E*_*μ*_) was computed for each segment using the following equation:

(1) $$E_{\mu }=\frac{\left|H\left(x\right)\right|}{N},$$

where |*H*(*x*)| is the absolute of the Hilbert transform [19] of iEEG segment and *N* = 640 is the number of samples in each iEEG segment. The segments with artifacts were identified from the other EEG segments by applying a predetermined threshold (Th = 0.6) after normalizing *E*_*μ*_ within the interval [0  1]. Threshold estimation is crucial since it is important not to label a seizure segment as a segment with movement artifacts. The threshold was determined by setting up a condition. The condition is the average absolute envelope of a segment has to be greater than the maximum of average amplitude of seizure segment to be considered as segment with artifacts. Therefore, it was confirmed that no seizure activities were falsely rejected as movement artifacts.

#### 2.2.3. Filtering of EEG

All iEEG segments were band-pass filtered between 0.5 Hz to 100 Hz using a 4th-order digital Butterworth filter to mitigate high-frequency noise and low-frequency artifacts. The iEEG segments were then notch filtered to remove 50 Hz power line noise.

### 2.3. Feature Extraction

Time domain, frequency domain features, and entropy-based features were extracted from iEEG segments. The four features used in this study were average amplitude, rhythmicity (coefficient of variation of amplitude), dominant frequency, and entropy. These features are known to contain the most discriminant information for detecting seizure events [7–12, 18]. Features extraction methods are described briefly in the following sections.

#### 2.3.1. Average Amplitude

Average amplitude (AVA) is a good measure for temporal evolution of partial seizures [7, 10, 18, 26]. During partial seizures, iEEG signals show rhythmic activity with a repetition frequency between 3 and 30 Hz [18, 26]. Therefore, to compute average amplitude, iEEG segments were first high-pass filtered above 3 Hz to remove low-frequency noise [18]. Then, a peak detection algorithm based on the zero-crossings of the first derivative of iEEG signals was used to detect peaks [18]. The amplitudes of the peaks were computed by taking average of the amplitudes of their half waves. Finally, the average amplitude (*μ*_amp_) was computed by taking the average of the amplitudes of the detected peaks [18, 26].

#### 2.3.2. Rhythmicity

Coefficient of variation of amplitude (CVA) is a measure of rhythmicity or regularities of ictal activities [18, 27]. During seizure evolution, the regularity of the amplitude of EEG tends to increase slowly; this increase is characterized by the CVA [27]. In case of partial seizures, the signals exhibit strong rhythmic characteristics which likely to have regularity in amplitude [27]. The coefficient of variation (CVA) quantifies the increased regularity observed during partial seizures [18, 27]. The CVA is defined as the ratio of the standard deviation of absolute amplitude to the mean absolute amplitude as [18]

(2) $$\delta_{\text{CVA}}=\frac{A_{\sigma }}{A_{\mu }},$$

where *A*_*σ*_ is the standard deviation and *A*_*μ*_ is the mean of each iEEG segment [18, 27].

#### 2.3.3. Entropy

Entropy is a measure of “irregularity” or “uncertainty” and was initially introduced by Shannon in 1948 [28]. The Shannon entropy (*η*) is computed as

(3) $$\eta =-\underset{k}{\sum }p_{k}\log p_{k},$$

where *p*_*k*_ are the probabilities of a datum in bin *k* [18, 28]. Approximate entropy (ApEn) introduced by Pincus and Goldberger [29] is more appropriate to compute the entropy for short and noisy time series data. A low value of the entropy indicates that the time series is deterministic, whereas a high value indicates randomness. Therefore, a high value of entropy indicates the irregularities in the iEEG data. To compute ApEn, it is required to determine a run length and a tolerance window to measure the likelihood between runs of patterns [29, 30]. The tolerance window *r* and embedding dimension are the two important parameters in computation of ApEn. In this study, Sample Entropy (SampEn) which is a variant of approximate entropy to quantify entropy of iEEG was used considering its robustness over ApEn [29, 30]. Sample Entropy is the negative natural logarithm of an estimate of the conditional probability that segments of length *m* that match pointwise within a tolerance *r* also match at the next point [18, 30]. This measure is a useful tool for investigating dynamics of biomedical signal and other time series.

#### 2.3.4. Dominant Frequency

Dominant frequency (*f*_Δ_) is defined as the peak with the maximum spectral power in the power spectrum of a signal [18]. This feature is particularly important in distinguishing ictal activities from interictal activities by quantizing the frequency signature information mostly found in partial seizures. This is characterized by a high-frequency activity at seizure onset and a low-frequency activity at the end of the seizures [18, 26]. In this study, parametric spectrum estimation method, autoregressive modeling (AR) approach, was used to estimate the spectral frequency band of the short EEG segments. The AR model order was chosen according to Akaike information criterion (20 in this study) [31]. The Burg method was used for computing the AR coefficients for short EEG segments [32]. Then, the spectral power of a given segment is estimated using these AR coefficients. For every spectral peak, the spectral frequency band was defined as [*f*_*l*_ and *f*_*h*_] where *f*_*l*_ and *f*_*h*_ are frequencies at rising and falling slopes of the peak with half the amplitude of the peak [10, 18]. The frequency of the peak with maximum spectral power is considered as the dominant frequency for the given segment [18].

### 2.4. Fuzzy Rule-Based Detection

#### 2.4.1. Design of Fuzzy Inference System

**Figure 1:**
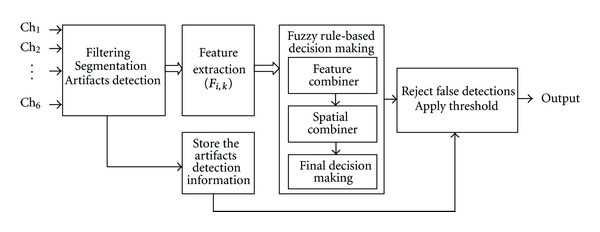
Block diagram of seizure onset detection system. The system comprises of preprocessing, feature extraction, decision making, and postprocessing stages.

In this study, we designed a multistage fuzzy rule-based system [33, 34] for seizure onset detection. Decision making was performed in three steps. We utilized the information obtained in spatial, temporal as well as feature domain to make the final decision. Therefore, the fuzzy system was comprised of three subsystems: (1) feature combiner, (2) spatial combiner, and (3) final decision making. Figure 1 shows the block diagram of overall system which includes preprocessing, feature extraction, fuzzy rule-based decision making, and postprocessing.

 Four features (*F*_*i*,*k*_,   where  *i* = 1,2, 3,4  and  *k* = 1,2,…, 6) were feed into the first fuzzy subsystem which is adaptive in nature (feature combiner): entropy (*F*_1_: ENY), dominant frequency (*F*_2_: DMF), average amplitude (*F*_3_: AVA), and coefficient variation of amplitude (*F*_4_: CVA). The second fuzzy subsystem (spatial combiner) was used to select four specified channels and combine the feature output from first fuzzy subsystem across channels. In final stage, another fuzzy subsystem was used followed by threshold parameter in order to classify an EEG segment as “normal” or “seizure.” The steps are discussed in detail in the following sections.

#### 2.4.2. Adaptive Fuzzy Inference System

**Figure 2:**
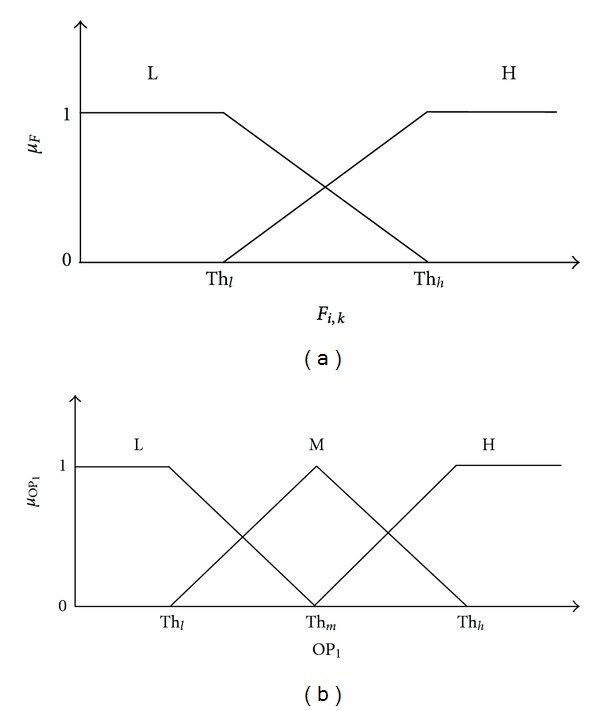
Triangular and trapezoidal membership grades assigned to the extracted features. (a) Fuzzy membership functions for feature inputs. (b) Fuzzy membership functions for feature output variable.

We have implemented the adaptive version of fuzzy inference system as described in the previous section. Four features were combined using a carefully designed fuzzy inference system. Before fuzzifying the feature variables, they were normalized into the interval of [0  1] using a min-max normalization method. Triangular and trapezoidal membership functions were assigned to the fuzzy input and output variables. Assigning membership function to the fuzzy input variables which are the features are extremely important and critical [35]. We utilized fuzzy clustering to adaptively estimate the parameters for membership functions [35, 36]. Fuzzy c-means clustering [35, 36] was applied to each of the feature to generate cluster center for two classes: “normal” and “seizure.” Then cluster centers were used to generate the membership function by placing the fuzzy sets at the corresponding cluster centers. Two membership functions or fuzzy sets were considered for each of the four input features: low (*L*:  *F*_*i*,*k*_ < Th_*h*_) and high (*H*:  *F*_*i*,*k*_ > Th_*l*_) as shown in the Figure 2(a). Th_*l*_ and Th_*h*_ were obtained from the corresponding cluster centers. This way membership functions were estimated adaptively based on the characteristics of the feature sets and the fuzzy system works adaptively. For the fuzzy output variable (OP_1_), three levels were assigned as high (*H*:  OP_1_ > Th_*m*_), medium (*M*:  Th_*h*_ > OP_1_ > Th_*l*_), and low (*L*:  OP_1_ < Th_*m*_) as shown in Figure 2(b). The values of threshold parameters chosen are Th_*l*_ = 0.3, Th_*m*_ = 0.5, and Th_*h*_ = 0.7. The OP_1_ is the final feature after combining the four features. We used triangular and trapezoidal membership functions for the ease of their implementation [18].

 The set of fuzzy rules for combining the features are listed in Table 1. Fuzzy logic has been utilized to combine this information obtained in feature domain using the first set of rules. The qualitative approach of fuzzy logic is specifically suitable to combine the four features and map them onto a final feature time series. The fuzzy output variable (OP_1_) will only be high “*H*” if and only if at least 3 feature input variables are high “*H*” and OP_1_ will be medium “*M*” if two feature input variables are high “*H*”. Rest of the times OP_1_ will be low “*L*” as shown in Table 2. Therefore, the imprecise boundaries of interictal EEG and uncertainty associated with features were addressed. For example, the behavior of rhythmicity alone may not hamper the performance of the overall system. More importantly, if any of the features is not able to detect subtle changes during seizure onset, a combination of the features using the fuzzy rules would we able to detect unless a seizure is missed due to nonspecific patterns. Similarly, spatial combination allows prioritizing the importance of in-focus channels due to their higher sensitivity to ictal activities.

#### 2.4.3. Spatial-Temporal Combination

**Figure 3:**
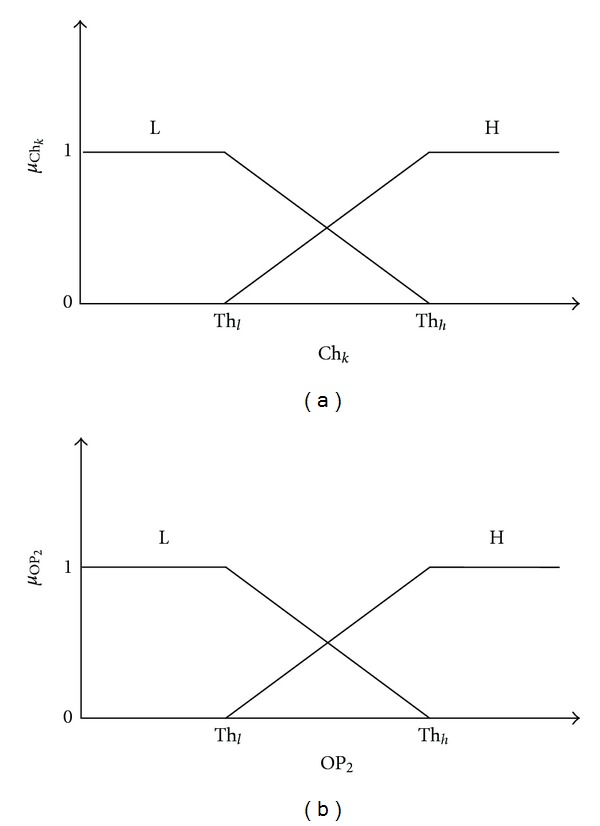
Trapezoidal membership grades assigned for combining across multiple channels to the extracted features. (a) Fuzzy input variable. (b) Fuzzy output variable. Two levels: high (H) and low (L) were considered.

For spatial combination, trapezoidal membership functions were assigned to the fuzzy inputs and output variable (Figure 3). Two levels were considered for both the input (Ch_*k*_ where *k* = 1,2, 3,4) and output (OP_2_): low (*L*:  *F*_*i*,*k*_ < Th_*h*_) and high (*H*: *F*_*i*,*k*_ > Th_*l*_). Three channels in epileptogenic zone (Ch_1_, Ch_2_, and  Ch_3_) were combined with one channel chosen from remote area (Ch_4_). These four channels were combined using another set of fuzzy rules based on experts' reasoning (Table 1). The criteria was set based on the information that the channels in seizure onset area is more sensitive in detecting changes in EEG comparing to those from remote area [1–3]. It is expected that in-focus channels will detect earliest changes in EEG. In order to minimize the detection latency, we considered all three in-focus channels in drawing up the rules for spatial combination. However, there are interactions between different channels location in brain. Therefore, to have modularity of the detection algorithm we have also included one channel from remote area. The set of fuzzy rules for combining the final feature output (OP_1_) across channels are listed in Table 2.

In final decision stage, averaging was performed for 5 consecutive segments using moving average method. At the final stage, another fuzzy inference subsystem was utilized to combine channel combination (OP_2_) and segment average (SA) information. Four rules were defined for mapping onto an alarm output space for preliminary decision making as shown in Table 3.

#### 2.4.4. Fuzzy Implication and Defuzzification Methods

The Mamdani-minimum implication operator was used for fuzzy inference and centroid defuzzification method was used to defuzzify the fuzzy output (FOP) variables [33, 34].

### 2.5. Postprocessing

#### 2.5.1. Artifacts and False Detections Rejection

Before making the final decisions, the system scans each iEEG segments for artifacts. In artifacts detection step, segments with artifacts were identified and the information was stored to be used in postprocessing step. False detections caused by artifacts were filtered in this step. iEEG segments corrupted with artifacts were assigned a value of “0” which leaves the probability of detection to zero too. We have performed further analysis on false detections and labeled the false detection rate as uninteresting and interesting [17]. The uninteresting false positives are mostly of short duration and caused due to residual artifacts and large amplitude rhythmic activities. We have rejected these short-length false detections by setting minimum length detection criteria [18].

#### 2.5.2. Threshold Parameter

We applied a threshold procedure for final decision making. Whenever the alarm “SZ” crosses the threshold, a seizure event was detected. Each segment was assigned probability value of “0” for normal segment and “1” for seizure segment.

### 2.6. Performance Evaluation Parameters

#### 2.6.1. Sensitivity

Since the objective of the system is to detect seizure onsets, sensitivity is an important statistical measure for event-based performance evaluation. It measures the ability of a system to detect seizure correctly. It is the measure of true positive rate and defined as the ratio of the number of correctly detected seizure onsets to the total number of seizures [4, 12, 15]. It is expressed in percentage as follows:

(4) $$\text{Sensitivity}=\frac{\text{TP}}{\text{TP}+\text{FN}}\times 100,$$

where  TP  and  FN  are defined as follows.

**True Positive (TP)**

The system detects a seizure that was annotated as seizure by the expert.

**False Negative (FN)**

The system misses a seizure that was annotated as seizure by the expert.

#### 2.6.2. False Detection Rate

False detection rate (FDR/hour) is another important parameter for the system performance evaluation [18]. It was computed by counting the false positives and divided by the total data length analyzed in the experiment for a given patient. To be successfully implementable in clinical settings, FDR should be considerably low so that neither the patient nor the caregivers have to wait too long under false alarms. However, usually it is better to detect the onset patterns with longer detection latency rather than missing them.

#### 2.6.3. Detection Latency

Detection latency is the time delay between the system detected seizure onset and clinical seizure onset identified by experts [9–12, 18]. Detection latency was computed as the difference between the clinical seizure onset (expert detected seizure) and system-detected seizure onset [12, 18]. For an automatic detection algorithm or in case of early detection, the detection delay time is expected to be considerably low or negative for early detection.

## 3. Results

### 3.1. Seizure Onset Detection

#### 3.1.1. Changes in Characteristics Features

**Figure 4:**
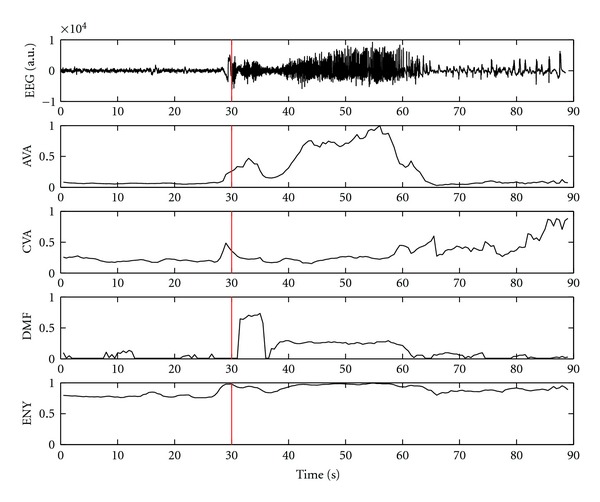
Seizure evolution profile: (a) Top subplot: an example of a seizure evolution in iEEG. (b) Bottom four subplots: corresponding changes in characteristics features: Average amplitude (AVA), coefficient of variation of amplitude (CVA), dominant frequency (DMF), and entropy (ENY). Seizure onset is marked by red vertical line. Early electrographic changes are visual in three of the four features.

Before designing the fuzzy logic system, visual inspection was performed to identify the types of changes in characteristics features at the time of seizure onset as well as offset. In most cases, the values of average amplitude increases after a few seconds on seizure onset. The values of rhythmicity gradually increase during seizure onset followed by a decrease to a minimum then return to the interictal baseline level few seconds prior to seizure offset [18]. In case of partial seizures, frequency activity increases right after the seizure onset up to a peak then gradually decreases to a low-frequency activity. Entropy values showed increase which reaches the maximum after a few seconds of seizure onset and fall down to interictal baseline at seizure offset. This means the complexity of signal increases during seizure. However, it does not increase to maximum right after the onset [18]. In some patients the electrographic changes are identified before clinical onset. Such a seizure evolution profile and the behavior of the characteristics features are shown in Figure 4 (patient 9).

### 3.2. Threshold Estimation

A threshold procedure was used to make final decision and assigning probability value of “1” to ictal iEEG segment and “0” to normal iEEG segment. The threshold procedure was applied to preliminary results obtained at the output of final fuzzy subsystem where the spatiotemporal combination was performed. The threshold parameter was optimized in a patient-specific way. The setting was optimized prioritizing higher sensitivity and lower false detection rate. It was determined by plotting the histogram of alarms generated for each patient. We used threshold values outside two standard deviations above mean. The range was two to six standard deviations above mean.

### 3.3. False Detections

For all patients, all the false positives less than 9.5 s were rejected except for patient 18 where the minimum length criteria was lowered to 4 s due to the unusual short length of one seizure onset pattern. After rejecting unusual short-length false positives, the system yielded average false detection rate of 0.26 per hour.

### 3.4. Performance Evaluation

A total of 112.45 hours of iEEG dataset having 56 seizures were used for system performance evaluation. Out of 56 seizures analyzed, the system correctly detected 54 seizures, whereas 2 seizures were missed. Therefore, the overall sensitivity achieved was 95.8%; the false detection rate was 0.26/hour, and average detection latency was 15.8 seconds.

**Figure 5:**
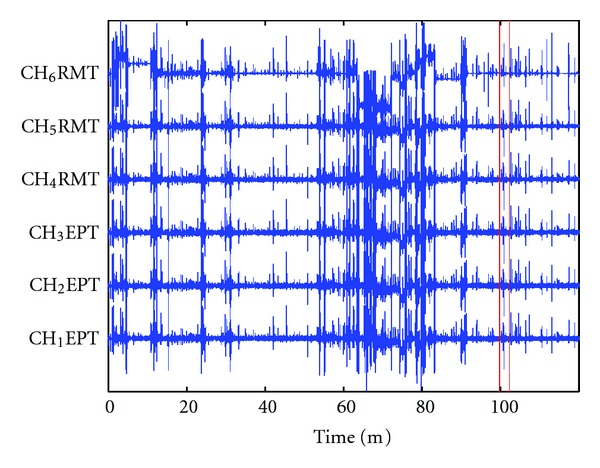
Seizure evolution profile in iEEG obtained from patient 10. Seizure onset and offset times are marked by red vertical lines, respectively. Acronyms: CH_1_EPT: Epileptic channel 1, CH_4_RMT: Remote channel 4.

 The data from patient 10 (of FSPEEG database) was discarded from the analysis due to the excessive presence of electrode movement artifacts based on the information obtained from the FSPEEG that in several occasions the measurement was exceeded, electrode box was disconnected, and reconnected as shown in Figure 5.

 Event-based sensitivity is reported in percentage. A seizure onset is considered as an event to detect. The average detection latencies are listed in seconds. Short-length false detections could also be reduced using a median filter or considering spatial criteria. The median filtering approach was tried but it has been seen that it falsely rejects some true detections which are unusually of short lengths. Also, it affects the detection latency. To address this, we utilized a postprocessor to minimize the uninteresting false detections which are significantly shorter in length then average seizure duration for each patient as described in postprocessing section. The overall results are presented in Table 5.

## 4. Discussions

### 4.1. Performance Comparison with Other Methods

Our method yielded average sensitivity of 95.8% with 0.26/h false detection rate. The average detection latency achieved was 15.8 seconds as shown in Table 5. The algorithm was developed in an unsupervised approach. We did not include the seizure free interictal data for evaluation purpose since there is no training involved. The dataset we used was constructed from the “ictal” data files from Freiburg project which have seizures with at least 50 minutes of preictal data and postictal data with no specified duration. Therefore, the false detection rate per hour is little higher comparing to other methods in the literature but reasonable considering the evaluation dataset.

Till date, many algorithms for epilepsy and seizure detection have been developed with different degrees of success [2–18]. Here, we have discussed briefly some of these methods providing a scope of comparison with our method. In a recent study, Zhang et al. [17] proposed an automatic patient-specific method for seizure onset detection using a novel incremental learning scheme based on nonlinear dimensionality reduction. Feature sets were extracted using continuous wavelet transform (CWT) [17]. Considering computation time and resources, the choice of discrete wavelet transform might have been better. Their method was evaluated on iEEG recordings from 21 patients obtained from Freiburg project with duration of 193.8 hour and 82 seizures. They have reported average sensitivity of 98.8% with 0.25/h interesting false positive rate and average median detection delay of 10.8 s. Aarabi et al. [18] introduced a fuzzy rule-based system for epileptic seizure detection which yielded sensitivity of 98.7% and false detection rate of 0.27/h with detection delay of 11 s. In this paper, different thresholds were used for different patients and a postprocessor was utilized to reduce the false detections in two steps. First short-length detections (less than 5 s) and artifacts were rejected. Secondly, two consecutive detections were unified given that they are less than a predefined minimum time interval (set to 30 s) [18]. Chan et al. [6] presented a novel patient-specific algorithm for seizure onset detection and accurate onset time determination. The algorithm extracts spectral and temporal features in five frequency bands within a sliding window and the feature windows were classified as containing or not containing a seizure onset using support vector machines (SVMs) [6]. Support vector machine is a popular classification paradigm for epileptic seizure detection and prediction being used by many researchers in this area. In order to accurately localize the seizure onsets in time, the method makes use of clustering and regression analysis [6]. Therefore, their algorithm yielded precise detection in time as reported in five of the six patients, at least 90% of the latencies are less than 3 s resulting median detection latency less than 100 ms with standard deviation less than 3 s [6]. However, the method utilizing user-adjustable parameters allow tuning to achieve high detection sensitivity, low false positive rate, and low detection latencies. Standard cross-validation performance measures resulted sensitivities in the range of 80% to 98% and false positive rates from 0.12 to 2.8/h [6]. Gardner et al. [5] presented a detection latency which is negative in time (−7.58 s) however with a higher false detection rate of 1.56 false detections per hour. Their system was evaluated on sample of 29 ictal and 41 interictal epochs and achieved 97.1% sensitivity [5]. Grewal and Gotman [12] proposed an automatic warning system with high sensitivity and low false alarm rates for clinical use. The system required training and was tested on locally recorded dataset yielding 89.4% sensitivity with false detection rate of 0.22 per hour and mean detection latency of 17.1 seconds with user tuning [12].

 The performance of our system is very much comparable to the other methods. It may not outperform the other methods in terms of all the performance measuring parameters. However, considering less mathematically complex design and lesser number of tuning parameters we have achieved similar results to other methods and in some cases better performance in terms of one or two performance measuring parameters.

### 4.2. Motivation and Advantage of Using Fuzzy Logic

The motivation behind our fuzzy rule-based approach is that fuzzy logic uses a much simpler rule-based design using natural language. Clinical neurologists mostly look at different features of seizure onset patterns as well as different channels to identify a seizure correctly. This is however complex to model mathematically and implement in computer programs. Fuzzy logic on the other hand provides a simpler design of approximate reasoning which can mimic human reasoning efficiently. We have developed our method in such way to mimic the experts' reasoning in detecting seizure onset patterns. Furthermore, the system provides a possibility of lowering the detection latency by incorporating more sensitive features.

 Fuzzy logic has been widely used in many signal processing and pattern recognition applications [33, 34]. Fuzzy rules can be defined using experts' knowledge for decision making which are simpler to implement and modular as well. Increasing the number of rules one can increase the accuracy of the model. Processing speed can also be improved significantly with less complex mathematical analysis and modeling. Moreover, fuzzy logic is a useful method for nonlinear input-output mapping which is effective in seizure detection or early detection applications. Other popular methods such as artificial neural networks and support vector machines require training, complex mathematical analysis, and modeling. In this study, we utilized adaptive version of fuzzy logic system with a novel approach of combining information in feature as well as spatial and temporal domain. A comparison of performance of adaptive fuzzy logic system is shown over conventional hard threshold-based methods and nonadaptive fuzzy system in Table 6. Nonadaptive fuzzy system is where the membership functions were generated in a heuristic way. Adaptive fuzzy system clearly outperforms other methods by demonstrating better performance in terms of better sensitivity and significantly reduces false positive rates.

## 5. Conclusions and Future Work

In this paper, we presented a robust method of detecting seizure onset using adaptive fuzzy logic system. Considering significant progress in the area of automatic seizure detection, we mainly focused in designing a seizure onset detection system in order to study the possibility of warning the patient or caregiver early in time. We also demonstrated the applicability of fuzzy logic in early detection or prediction system by comparing the performance improvement over conventional hard threshold system. The adaptive version of the fuzzy system is capable of tuning some of the system parameters in a patient-specific way. This is crucial given the wide varieties of seizure types as well as stereotyped evolution and onset patterns in a given patient.

 The algorithm was developed in MATLAB and tested offline. For fuzzy c-means clustering, we used MATLAB function *fcm* with default parameters (exponent of the partition matrix U: 2.0 and maximum number of iterations: 100). The overall system makes use of temporal information of iEEG as well as spatial information, since there are interactions between the channels, obtained from three channels located on seizure focus. Given the promising result of this study, adaptive fuzzy algorithms could provide a robust method for designing an early detection or prediction system. The possibility of reducing the detection latency for early detection largely depends on the features sensitivity to detect the preictal changes. Therefore, the performance of the system could be improved by incorporating feature selection techniques or choosing features that are sensitive to earliest electrographic changes in EEG.

In future work, more analysis will be performed based on the findings of this study to relate the iEEG findings with brain mechanisms. We will be looking into some of the specific patterns found in this study as well as study the data from several patients where interictal activities are stronger (patients 1, 2, 13, 18, and 21). Also, we will study the data patterns where there are excessive presence of interictal spikes or large amplitude rhythmic activities (patients 8, 10, and 18). These findings have been previously reported in the literature as well [17] and might be the reason for longer detection latencies in patients 8 and 21.

False alarms could be reduced significantly by considering the recent developments in artifacts detection techniques over standard threshold-based methods. In the present study, standard threshold-based movement artifacts detection method was used to avoid computational complexity. In future studies, a machine learning-based movement artifacts detection method will be included. Finally, we will attempt implementing a similar approach with relevant features in early detection of epileptic seizures and eventually targeting the goal of prediction.

## Figures and Tables

**Table 1:** Fuzzy rules for combining features.

*F*_1_	*F*_2_	*F*_3_	*F*_4_	OP_1_
H	H	H	H	H
H	H	H	L	H
H	H	L	H	H
H	L	H	H	H
L	H	H	H	H
H	H	L	L	M
H	L	L	H	M
L	L	H	H	M
L	H	L	H	M
L	H	H	L	M
H	L	H	L	M
H	L	L	L	L
L	H	L	L	L
L	L	H	L	L
L	L	L	H	L
L	L	L	L	L

*F*_1~4_: Feature 1 to Feature 4;  OP_1_: Output 1.

**Table 2:** Fuzzy rules for combining channels.

Ch_1_	Ch_2_	Ch_3_	Ch_4_	OP_2_
H	H	H	H	H
H	H	H	L	H
H	H	L	H	H
H	L	H	H	H
L	H	H	H	H
H	H	L	L	H
H	L	L	H	H
L	L	H	H	H
H	L	H	L	H
L	H	L	H	H
L	H	H	L	H
H	L	L	L	L
L	H	L	L	L
L	L	H	L	L
L	L	L	H	L
L	L	L	L	L

Ch_1~4_: Channel 1 to Channel 4; OP_2_: Output 2.

**Table 3:** Fuzzy rules for mapping onto an alarm output space.

OP_2_	SA	SZ
H	H	H
H	L	M
L	H	M
L	L	L

OP_2_: Output 2; SA: Segment average; SZ: Final output.

**Table 4:** Summary of the iEEG data selected for analysis, including patient number, total data length, gender, age, seizure type, seizure origin, the number of analyzed seizures, and average seizure duration per patient. Acronyms: SP: simple partial seizure, CP: complex partial seizure, GTC: generalized tonic-clonic seizure, F: female, M: male.

Patient	Data length (hour)	Gender F: female M: male	Age	Seizure type	Seizure origin	Number of analyzed seizures	Average seizure duration (seconds)
1	2.48	F	15	SP	Frontal	3	15.1
2	5.16	M	38	SP, CP, GTC	Temporal	2	107.97
3	5.10	M	14	SP, CP	Frontal	4	88.67
4	5.87	F	26	SP, CP,GTC	Temporal	3	86.46
5	3.81	F	16	SP, CP, GTC	Frontal	2	14.72
6	4.13	F	31	CP, GTC	Temporo/Occipital	2	78.6
7	3.91	F	42	SP, CP, GTC	Temporal	2	70.71
8	3.49	F	32	SP, CP	Frontal	2	163.72
9	8.83	M	44	CP, GTC	Temporo/Occipital	5	113.02
11	4.92	F	10	SP, CP, GTC	Parietal	3	195.83
12	7.87	F	42	SP, CP, GTC	Temporal	4	55.06
13	3.92	F	22	SP, CP, GTC	Temporo/Occipital	2	158.3
14	4.91	F	41	CP, GTC	Frontotemporal	3	264.95
15	5.92	M	31	SP, CP, GTC	Temporal	2	202.39
16	9.83	F	50	SP, CP, GTC	Temporal	4	138.94
17	14.59	M	28	SP, CP, GTC	Temporal	5	86.16
18	1.96	F	25	SP, CP	Frontal	1	13.64
19	5.92	F	28	SP, CP, GTC	Frontal	2	15.32
20	6.87	M	33	SP, CP, GTC	Temporoparietal	3	122.51
21	2.96	M	13	SP, CP	Temporal	2	79.04

Total	112.45	7 M/13 F	29.9	—	—	56	103.56

**Table 5:** Summary of the results: sensitivity in percentage, false detection rates per hour, and average detection latencies in seconds.

Patient	No. of seizures	Data Length (h)	SEN (%)	FDR/h (uninteresting)	FDR/h (interesting)	Detection Latency (s)
1	3	2.48	66.67	4.4	0.40	7.21
2	2	5.16	100	2.52	0.39	25.03
3	4	5.10	75	0.19	0.19	8.72
4	3	5.87	100	1	0.17	27.43
5	2	3.81	100	0.26	0.26	23.97
6	2	4.13	100	0.72	0	12.64
7	2	3.91	100	1.02	0	17.46
8	2	3.49	100	1.43	0.57	55.46
9	5	8.83	100	1.24	0.34	−24.92
11	3	4.92	100	1.01	0.40	−6.84
12	4	7.87	75	2.16	0.50	21.04
13	2	3.92	100	0.51	0	−37.69
14	3	4.91	100	0.61	0.20	40.14
15	2	5.92	100	0	0	27.37
16	4	9.83	100	3.86	1.01	5.64
17	5	14.59	100	0.06	0	23.52
18	1	1.96	100	1.02	0	0.31
19	2	5.92	100	0.33	0	1.33
20	3	6.87	100	0.43	0.14	27.07
21	2	2.96	100	4.72	0.67	61.42

Total	56	112.45	95.83	1.37	0.26	15.81

**Table 6:** Performance of adaptive fuzzy system over single method with conventional hard threshold and nonadaptive fuzzy system.

Method	SEN (%)	FDR/h
Feature 1 (hard threshold)	96.25	1.93
Feature 2 (hard threshold)	93.75	3.62
Feature 3 (hard threshold)	98.75	1.16
Feature 4 (hard threshold)	84.17	1.98
Nonadaptive fuzzy system	91.49	0.35
Adaptive fuzzy system	95.80	0.26
